# Supplementary material for: Perinate and eggs of a giant caenagnathid dinosaur from the Late Cretaceous of central China
Source: Nat Commun. 2017 May 9;8:14952. doi: 10.1038/ncomms14952 (PMC5477524; doi:10.1038/ncomms14952)
Supplement: Supplementary Information — Supplementary Figures, Supplementary Tables, Supplementary Notes and Supplementary References [file ncomms14952-s1.pdf]

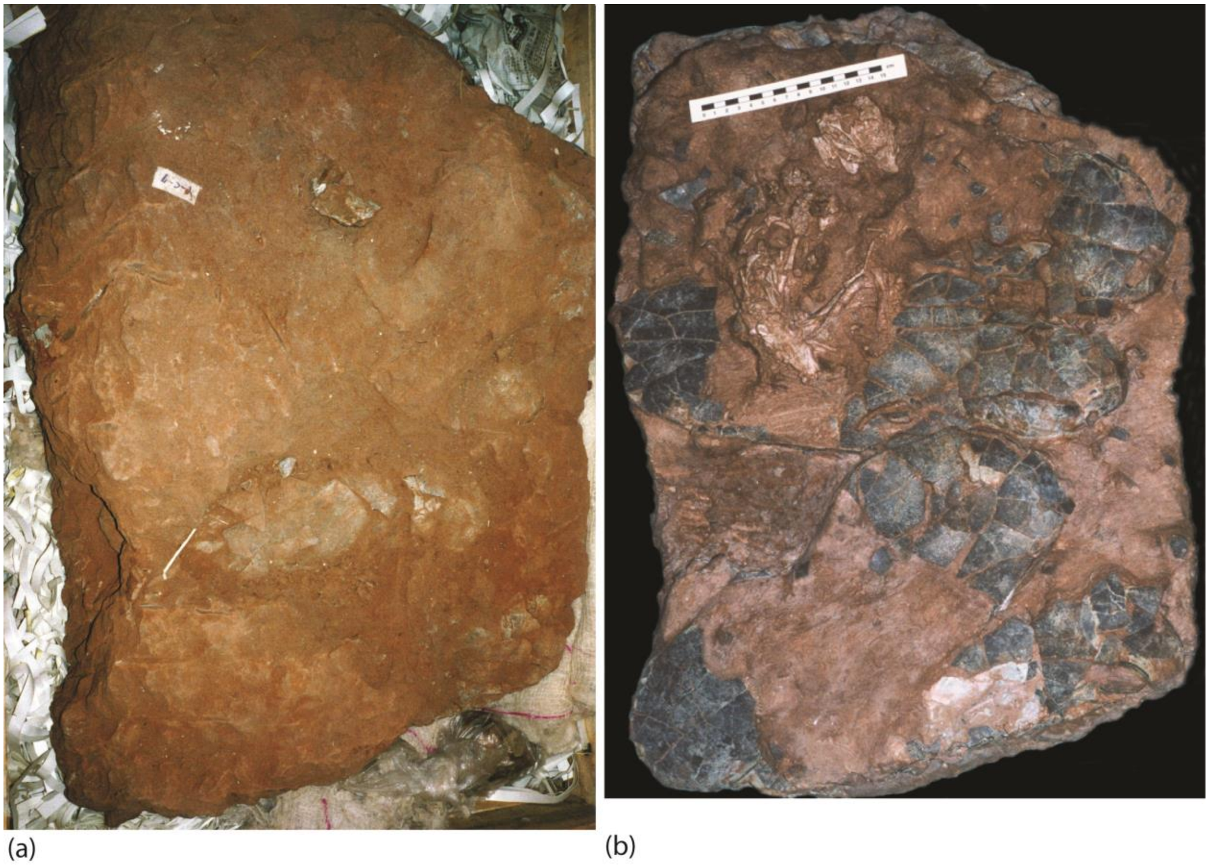

Supplementary Figure 1 | Photographic comparison of unprepared and prepared block of *Beibeilong sinensis (*HGM 41HIII1219). (a) Unprepared block in crate in China in 1993 (photo courtesy of Florence Magovern, The Stone Company). (b) The skeleton and eggs were exposed during preparation by Charlie Magovern of The Stone Company in Colorado (photo by Darla K. Zelenitsky).

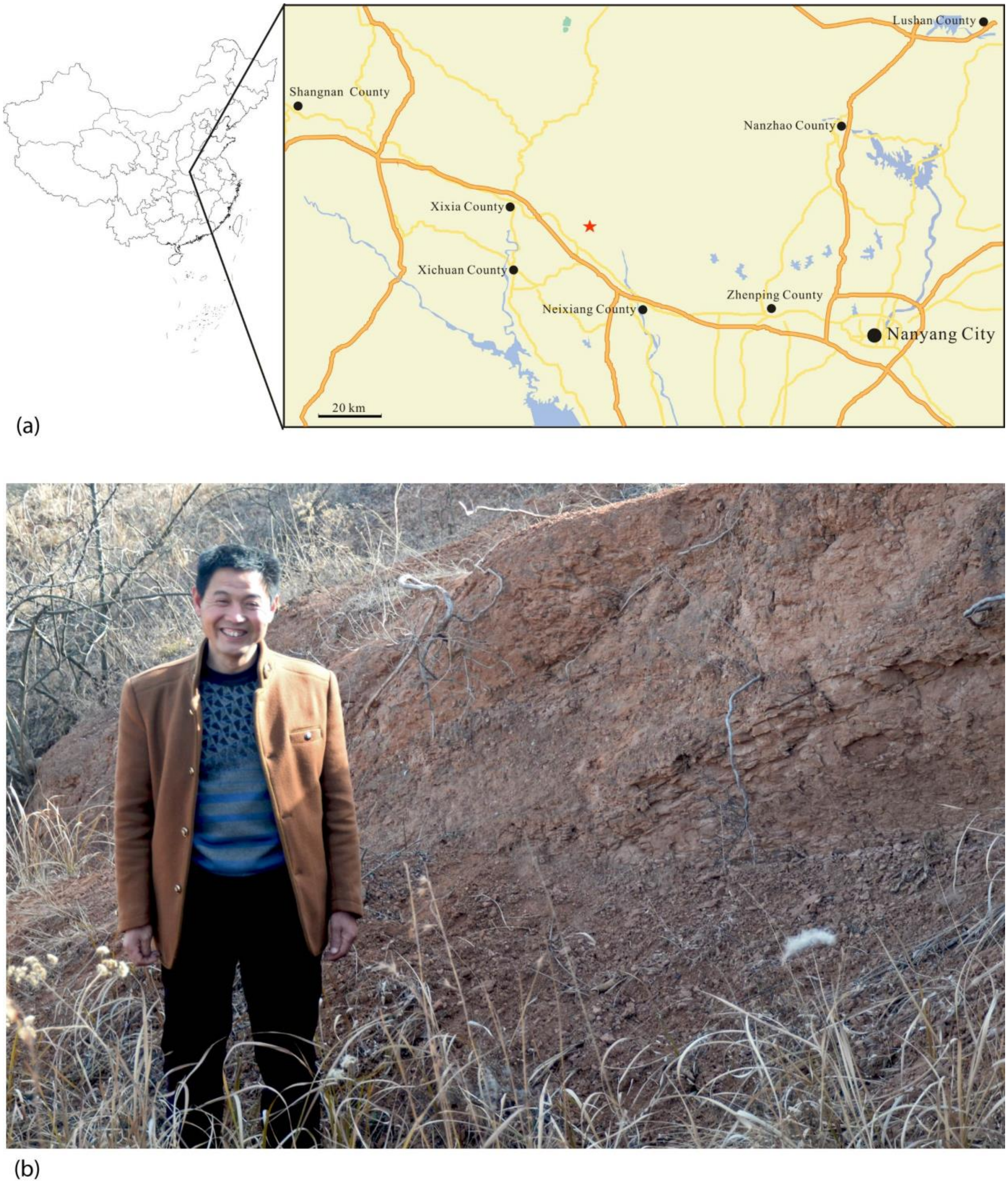

Supplementary Figure 2 | Locality information for *Beibeilong sinensis* (HGM 41HIII1219). (a) Map of the fossil locality near Nanyang City, Henan Province, China. Star represents the fossil site where the Baby Louie specimen was collected in 1992-1993. (b) Mr. Zhang Fengchen at the field locality in 2015 where the Baby Louie specimen was collected in the Xixia Basin of Henan Province (photo by P.J. Currie).

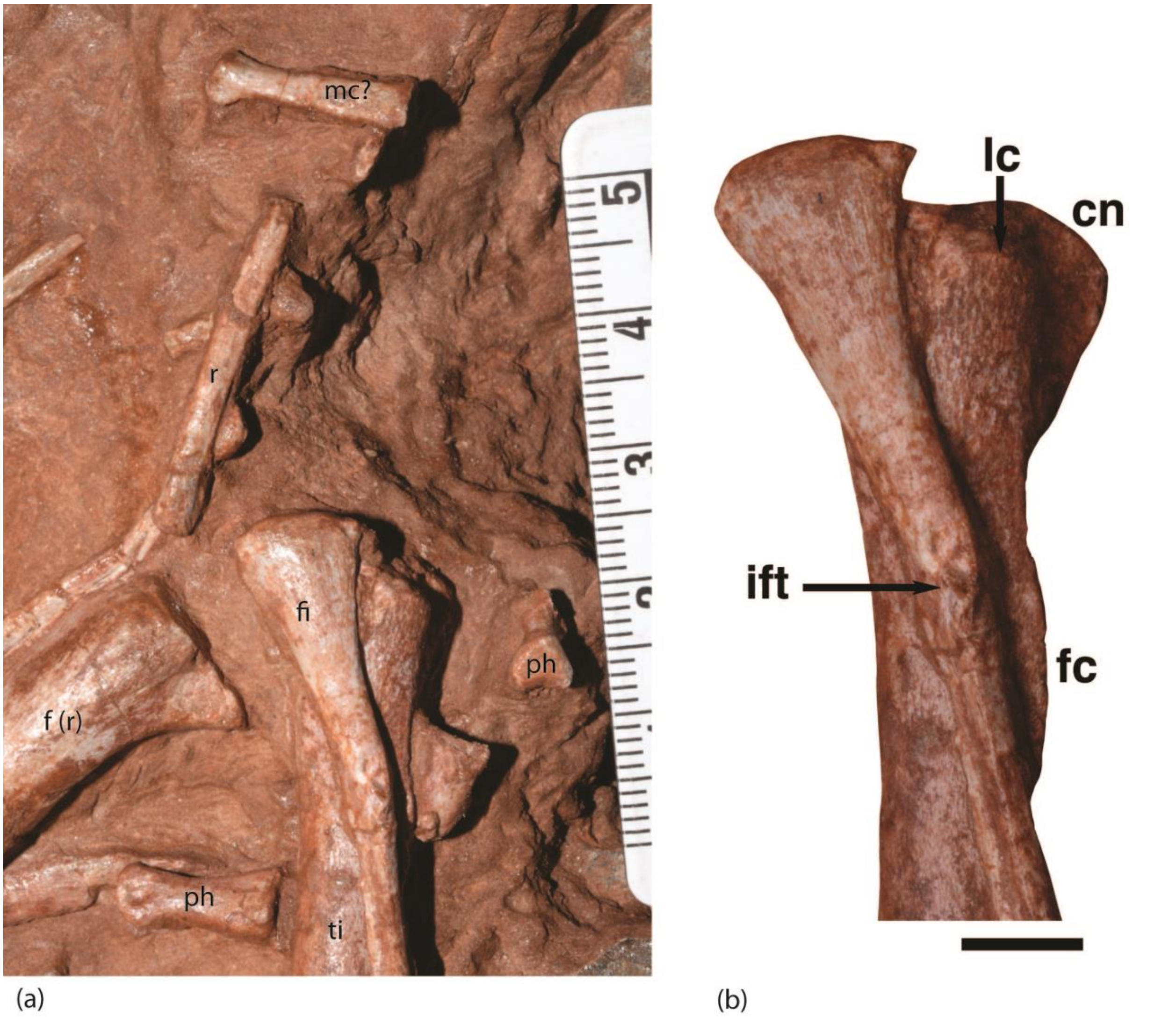

Supplementary Figure 3 | Photographs of the hindlimb region of *Beibeilong sinensis* (HGM 41HIII1219). (a) Bones identified in the region of the right knee. Scale bar in cm. (b) Features on proximal ends of right tibia and fibula. Scale bar is 5 mm. Abbreviations: cn, cnemial process; f, femur; fc, fibular crest; fi, fibula; ift, iliofibularis tubercle; lc, lateral (fibular) condyle; mc, metacarpal; ph, phalange; r, rib; ti, tibia.

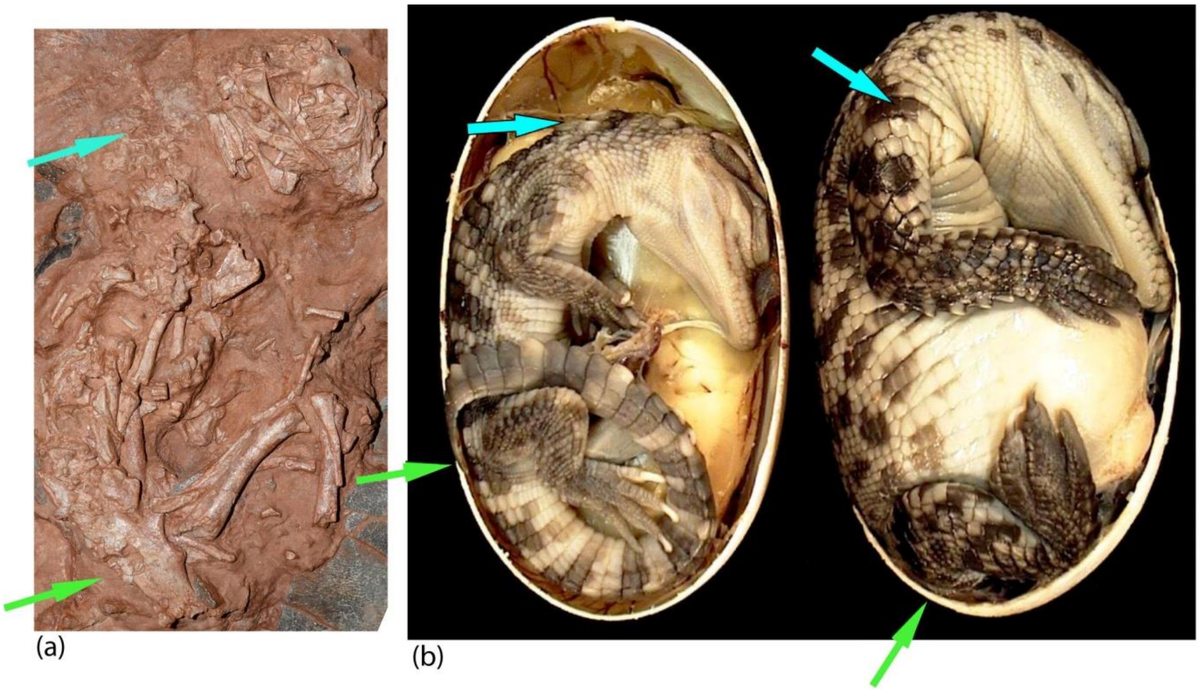

Supplementary Figure 4 | Comparison of embryonic pose of *Beibeilong* (HGM 41HIII1219) (a) with that of the Siamese Crocodile (b). Neck and hip orientation indicated by blue and green arrows, respectively. The neck region is slightly ascending in the *Beibeilong* skeleton, and the pelvis and hindlimbs project above what would have been the base of tail. *Beibeilong* embryo appears more similar to 51 day crocodile embryo (left) than to near-hatching 78 day embryo (right). This approximation of the prehatching stage of the *Beibeilong* embryo suggests that it may have died before completing three fourths of *in ovo* development.

Supplementary Table 1 | Measurements of eggs in specimen (HGM 41HIII1219)

Egg #	Length (mm)	Width (mm)	Elongation Index	Comment
1	450	120+	3.75:1 -	UL
2	415+	155-	2.76:1 +	UL, bone
3	400	152-	2.63:1 +	UL
4	340++	inc		UL, bone
5	435	cov		LL, bone
6	cov	cov		LL

Egg numbers from Figure 1 (the perinate skeleton is on eggs #3 and #4). Because of differential crushing, and the fact that none of the eggs have been prepared out completely, none of the measurements accurately indicate the original egg size (prior to burial). Although compressed, the eggs were up to 450 mm long with a width of about 150 mm at mid-length; the compressed width is probably somewhat greater that the original width. Abbreviations: bone, associated with embryonic bone; cov, covered by matrix; inc, incomplete; LL, lower level; UL, upper level.

Supplementary Table 2 | Measurements (in mm) of *Beibeilong sinensis* (HGM 41HIII1219).

Snout-vent length	380
Skull length (preserved)	66
Orbit length and height	24
Jugal length	34.5
Jaw length, estimate	65
Dentary length, estimate	32
Dentary height, maximum	18
Scapula, minimum shaft width	4.7
Metacarpal II	15.2
Ilium length	66
Ilium, height above pubic peduncle	26.7
Ilium acetabulum length	15
Pubis length	62.4
Femur length, estimate	75
Femur, transverse shaft width	8.8
Tibia, proximal width	15.6
Tibia, transverse shaft width	7.2
Fibula, proximal width	9.6
Pedal phalanx III-2	11.9
Pedal phalanx IV-2	6.1
Pedal phalanx IV-3	5.7

Supplementary Table 3 | Measurements (in mm) of various perinate oviraptorosaurs.

Specimen Number	Egg L	Femur L	mt III
CM-41	169		
HGM* 41HIII-0107	x	72	43.3
HGM* 41HIII-0301	180est	46	
HGM* 41HIII-1219	450	75	x
MPC-D110/971	x	x	x
NMNS-0015726-F02-embryo-0 l	174		32
HGM** 669	inc	65	41

Abbreviations: *HGM, Henan Geological Museum;**HGM, Hicksville Gregory Museum

Supplementary Table 4 | Character codings for each taxon in the phylogenetic analysis. Character matrix is from Funston and Currie[1].

Herrerasaurus_ischigualastensis

00000000000000000000000000000000000000000000000000000000000000000000000000000
000000000000000000000000010?000000000100?0000101??00000000000000000100000000000
000000000?000?0000110010100??000??0000101??00??0000???00?1000?000000?00000??????
?000?0000?000?

Velociraptor_mongoliensis

00010000000001010010100101000100000000010000000010000100000000000000000000000000
0100001100000000000100101101111011010111011-110101111011100001111112010011100010
011000000000?0000000011000?0000??0010000100000000110011?000?00000000000000????
?00000000?000?

Archaeopteryx_lithographica

000?100000001011010011000100001?10002110?0?1??0020???00????0000001?1???100001021
000011-0100000020100??0101?00?110110012111001111121011011010111020101?0100?11012
00?0000?0?0?00000001000?0000??001?000000?010010?0?111??00?00000?0000?00??????000
00000?000?

Avimimus_portentosus

?0?????0?????0?0102020????10110??1?2121201??00?101111?1???1???????????01200111??01
?001?11000???21?21000110?110??0???2?02100111?1?????00000?210?100201010011112221O
0010000?0??????00?100100000?0000000??0??10110??11110????0?000?0?001010?111100000
00?00?1

Incisivosaurus_gauthieri

100110101000011?0111210011?01?00?0000?10010?0??200111?1?10?01??111?0101?21011102
0??00011??11?111011??00000
000100??????????0010000000000000???????1??????00???01000???????0???????????????00
0?

Beibeilong sinensis

10??????111000101?????1?01?0?0???1100?1??????11??????????????11?????????111111202?0?0
00111112????122???0??1??????????????????0?????????1011?20111?1??11111??000?0?1??10?0
?00???????000??00?0?00??00?101????00?1??????1???10??0?1?????????????0????0000?00?0

Microvenator_celer

??1111?2????1100
1????????????2???0??1?????????0????1?0011??1????0???112?11?112??011101????????0101???
001??????00??0000000?0??????0?1??1???0??1?0???1?????0??00??????????????????????

Caudipteryx_zoui
10?110?01??0?1110?0?21?01?10???0110001110100??????????????????0????????0??1111?2???
0001??????11?0220?????010????0?1??10???1011?1020100?001220110?021100???1000121??0
???0??0?00?001000?000?00000000?01000?0010???0101????0?00000?0000000?0??????000000
?000?

Caudipteryx_dongi
???
?????????????????01?0?????????????0010?1?20100?011?2011000211?????100012???????????
?00?001000?????????????????01?10?????0??1?????????????00000???????000000000????

Oviraptor_philoceratops
1??????????1?11?????21????????1??1000???0???????????????????????1?????????1?12122??10
011???1??1??22???0??1?????????????1???101101?211??????????????????????????????0???0010
01000000??0??1?????????????????0??????????0??11?0?0???0??00???0???????????????000?

Rinchenia_mongoliensis
11122?0111110111???1211111111?1111010111001111111?1111121?111111112111111111111212?0
11001111111121221110011?1010010102?12101000???????01101121101????111111??????110
1?001011????????00111??????00000001????????1?10???1110?0?0?0???????????????????????0
00?

Citipati_osmolskae
11?2221?1111011?11112111111?111?1111011111??????111????11?1?11111????1?11?111212201
1001111111111122????????1?????????????????0?101?2??????????????????????????????11011001
011?01010?0??1100000000000000100?????010100?01110?001000100?0?0??????00?0??00?00
0?

Zamyn_Khondt_oviraptorid
11222211111101111111211111111111111101111111111111111121111111111211111111111121220
11001111111121221110111?101001010210210101101121110100121100111211111111110001011
01?001011?0000010??110?0000000000?0100???00?1?100??11???001?0??00??00?????????????
??000?

Khaan_mckennai
1022111?1111011?1121211111111111?1100011100?111?111111??0??1?????1??????11111121210
1100111111111??1221110??1????00?0102??210??1101021110?000110001?1211111?1100010100
1100100100101001111110?000?0010000001000000?01?1100?1?10101000?0000100??????00000
000?000?

Conchoraptor_gracilis
1012111111110111112121111111111111110001110?1111111?11112111111111111211111111111?12?0

110011111111121221110111?2010010101112101111001211101000210001112111111110001010
1?????????110???1??11?????????????????0??????11?00?011?0?0000011?0??000?????00000000
?000?

Machairasaurus_leptonychus

???
???????????2???????????????????????????0012???111
110??????????????????????00???????????????????????????1????????????????????????

Nemegtomaia_barsboldi

1112011111101111112101111111?110001011011?111111?1??11?1111112111111111111212?1
1100111111?1121221110211?201????????????????????????10002??00?1????11?????????10111
101011?12?11??1111000000010000001000??0??1???1??11?1?1?0001?000??00??????????????0
00?

Heyuannia_huangi

?????????????????????????????????1??1??2121??100
1????1??????21???21-12????1?????121?11000002?20???01?1100111?1????????????????1?1?10
11112?2101?1?????????0?00000100?0?0?0101?00?1?11?0???0?00001????????000000000000?

Ingenia_yanshini

???????1???????????????11?????1?11000111?0111111???111??11?1??111?1???1111111212?011
0011111111112122?110011?2010010101012101100001202001001210001112111111111100010?01
1?1010111212101111100000000000000100?0000?1?100001?1??0???0?000010?00000000000
000?000?

Gigantoraptor_erlianensis

??1121111202011100
111112????1?2???????????0010?0??00?00000?11?1??1???????????1??1111111000?0???000101
0000???????????0???0001001110100???0??????1?????110??112?10??????????010?000??011?

Gigantoraptor_Mongolia

??2111??????21???
??001???????
?????????0011000?1????????????????????????1????01???????????????????????????

Caenagnathasia_martinsoni

??2101?1????120?
?????????????2?1002?1?0?1???10?1???????????0001???0
?????????????011?111?1????????????????????????????????0?????????0??????????????????

Caenagnathus_collinsi

??1021011102011200
1111111112??2??????????????1?????????????????????????????????????111101?001?????00010101

?1??????????11011210111111?????1????????01?0010??201??1?????????????0???0??000?

Leptorhynchos_elegans

???12111?1????1200
???????1????2???1111????0001???0?
??????0????0111121?10???????????1???????????0?????0??????1?????111100111111??1

Elmisaurus_rarus

????????????????????????????0???
???????????????0??10?????????2????1?????011?????????????????11?1???1111??0?????????00
0?10?0???????????????????11????1?????????????????????1??111111111110111120??11

Apatoraptor_pennatus

???0?1???121111102011100
1111111112??211002111?????????2?0111001001110111???101???????????101?????????000101
00010?10??????01?11?11111111210??????1?101?11??011??101?01????111?????????????1001

Chirostenotes_pergracilis

??112111110201120 0
1111121112??2????????111??????????10?????0????1??11??21101???111?1?1100110???000101
00?100101000??01111211111111112?1111????????1??010???01??1000??11110000110010 0110

Hagryphus_giganteus

???
???????????????????????????????????????0010??00
0010???????????????????????2????????????????????????????1?????00???????????????1?

Anzu_wyliei

?1??101100?1100??????????????????0?000??????1011??100?1??1??0?011?????1121011?02011
2001111111?121221110211?1?10010?02?02100010????1110???1?2?1?110211111111?????1?00
0010100000??01?0??011011211101111 1??1111?110101101?00101021201110???1??1????0????
?0000

Nomingia_gobiensis

???
???????????????00?1?01?00101020????????????????111011011111011111111????????????????
??????1?00????????????????????111???0???1???1????????1??10?????????????????????

Epichirostenotes_curriei

?????????01?010??????1?????????????????????????????110?1?1??0??????????????????????????
?????????????????2?1?111??1???2??????????????????????????????021????????????????????????
?????1???????????????????????111??0?0???1????????????????10??0??????????????????

Banji_long

11?210111???0111102121111111011??11101????0??11?1111????????1111121?0111??11?2121?

?000110??1?11?122?1???1111100
1011??????????110??????0?00000????????????????00?0?000?0???????0???????????????????

Ganzhousaurus_nankangensis

???11111?21?201100
1?????111???2?????????????0???0??0001????1?001?11
???????1????????0?001?10?0???????0???????????111???0??????????????000000000????

Jiangxisaurus_ganzhouensis

1??????????????????????11???????1?001????????11?????????????1??1??????111111??101?1?0
011?111????122??10?????????1???????1?1100?0?10????0???????????????????????????1?001?
10??2??????????????????0?00000?0?????????1?0??????????00??00????????????????????????

Nankangia_jiangxiensis

??11101?2?????100
????????????2??????1???1??1??0?0011001?????????011101111111211001111????????1101??0
???????1?00??11?????0?1???1????11???????01???11?0???0??0?00?????????????????????

Shixinggia_oblita

??
??????????????????1?2?1??1??????????????????????110000000?????1????1??????????????????
??????01???

Wulatelong_gobiensis

11?2211?11?1?1???02121?111?1??1?111001??00??11???????????????1111???????????????101?
???1??11????12???????1???100????2??????????1??????1100?211111121???????000?01???????
????0??????011????????????000????1????1?10?101?????????????0??0??0???????000000????

Yulong_mini

1020101110110110111121010111010?01000111010?111110110??0????????0?????11111112122
011001111111?12122?100??1????001?10??1??0000101?21010?110?10??0??0?1?0??11?001010
?11001011?11000?100110??????0?0000010???????1??????1110?01000??00???00?????0000000
00????

Supplementary Note 1 | Preservation of HGM 41HIII1219.

The specimen is a partial nest of eggs that is preserved in hard, red, fine sandy siltstone. When the specimen arrived in the United States from China, part of another egg had been attached onto the block to increase the price of the specimen; this was removed during preparation. Otherwise, there had been no tampering with the block, and all parts were solidly connected by undisturbed, unfractured and unglued matrix.

HGM 41HIII1219 is a small, semi-articulated skeleton (Baby Louie) lying across two adjacent eggs in the upper layer (Fig. 1). The anterior part of the skeleton rests on an egg (egg #4) in which the equatorial region and the outer polar region are missing on the upper side, whereas the posterior region of the skeleton lies on an adjacent egg in which a large (11 cm long) window of eggshell is missing in the equatorial to sub-polar region (egg #3). Fragments of eggshell close to the backbone of the skeleton have been inverted to expose inside surfaces, and presumably represent the shell that was removed when the perinate was removed or extruded.

A third egg lies at a roughly 45-degree angle to the other eggs, and is also missing a large window of shell from the equatorial to subpolar regions (egg #2). The third egg appears to have been partially displaced and covers the outer polar region of another egg (#1), which is subparallel with the first two (although partially separated from them by the inner polar end of the third egg). Several bones lie on top of this egg (#1), including several vertebral centra, rib fragments, a radius and an ulna. It is possible that these bones are part of Baby Louie, and had drifted 30 cm before burial of the nest and skeleton. Evidence of another possible skeleton is an unidentified limb bone protruding from inside one of the eggs (#5) in the lower layer of the nest.

The skull has collapsed into a horizontal layer of overlapping bones (Fig. 4a). The premaxillae and the front of the right mandible seem to have been destroyed postmortem, but before burial, by the activity of unknown osteophagous or boring insects. The right side of the skull has shifted dorsally in relation to the left, so the fused nasals are exposed in ventral view, and most of the bones from the midline and the left side of the skull are covered by the right side of the skull and jaw, and by matrix. The left dentary has drifted up to 4 cm away from the rest of the left jaw (Fig. 2). The front of the right dentary was broken and lost.

The left dentary, and elements of the neck, abdominal region, pectoral girdle and forelimbs are disarticulated (Fig. 2). Most seem to lie in a jumble in the abdominal region, which makes it difficult to identify or measure complete bones. The tail cannot be seen, but may lie underneath the rest of the body, or may have been lost through scavenging. The pelvic girdle and hindlimbs are present and mostly articulated, but the lower part of the right leg appears to have been destroyed by osteophagus or boring insects. Bones from the abdomen and the right pelvic girdle and leg obscure most of the left leg.

Borings and elongate burrows left by osteophagus or boring insects are associated with the

specimen, and the skeleton suffered postmortem insect damage to the ilium, the proximal end of the femur and other regions.

Supplementary Note 2 | Dentary proportions of *Beibeilong sinensis* (HGM 41HIII1219).

The height to width ratio of the dentary is 0.56 for *Beibeilong*, which compares most closely with the ratios in *Gigantoraptor* (0.56) and *Microvenator* (0.60). The distance between the anterior margin of the dentary and the front of the external mandibular fenestra is 23 mm, whereas the minimum height immediately behind the symphysial region is 11.3 mm. Again, the ratio between minimum dentary height behind the symphysis to length between the front of the jaw to the front of the external mandibular fenestra is closest to *Gigantoraptor* and *Microvenator* (all three are approximately 50%). Finally, a comparison of minimum to maximum dentary height produces a ratio in Baby Louie of 0.63 compared with 0.65 in *Gigantoraptor* and 0.59 in *Microvenator*. The minimum/maximum ratios in other caenagnathids and oviraptorids, regardless of age and absolute size, range between 0.23 and 0.56.

Supplementary Note 3 | History of *Beibeilong sinensis* specimen.

The history of the Baby Louie specimen is somewhat unusual for a specimen exported from China at the time. While not the case with this specimen, such fossils can be damaged beyond repair, their original locality information lost, or they disappear into private collections. For this particular specimen, its whereabouts was always known. From 1993 to 2001, it was at The Stone Company in Boulder, Colorado where it had been carefully prepared by Charlie Magovern. During its preparation, several paleontologists were made aware of the specimen and examined it. It was then on display at the Indianapolis Children's Museum from 2001 until it was repatriated to the Henan Geologic Museum in China in 2013. Additionally, the original locality and collector of the specimen fortunately could be traced back to a farm in the Xixia Basin of Henan Province, which is still yielding eggshells from the site.

Although unfortunately it took 20 some years to publish this important specimen, it was a very unusual specimen at the time and other specimens found subsequently had significant bearing on its study. In the early 1990s, the discovery of the specimen and other *Macroelongatoolithus* eggs were perplexing as the eggs/nests were gigantic compared to what could reasonably have been laid by known oviraptorosaur species. Furthermore, the highly unusual skulls of oviraptorosaurs were poorly known in perinate or juvenile form then, so identifications were not straightforward in an immature, disarticulated, and flattened skull. In the years following its discovery however, juvenile/hatchling oviraptorosaurs with unfused skull elements helped make sense of the bones in the perinate [2-4]. Finally the discovery of a giant caenagnathid (*Gigantoraptor*) in 2007 from northern China revealed that oviraptorosaurs species

capable of laying such large eggs existed[5]. A portion of the 20 years at least shed light on some initially puzzling aspects of this specimen.

Supplementary References

1. Funston, G. F. and & Currie, P.J. A new caenagnathid (Dinosauria: Oviraptorosauria) from the Horseshoe Canyon Formation of Alberta, Canada, and a reevaluation of the relationships of Caenagnathidae. *J. Vertebr. Paleontol*. **36**, e1160910 (2016).
2. Ji, Q., Currie, P.J., Norell, M.A., & Ji, S. A. Two feathered dinosaurs from northeastern China. *Nature* **393**, 753–761 (1998).
3. Makovicky, P. J., & Sues, H.-D. Anatomy and phylogenetic relationships of the theropod dinosaur *Microvenator celer* from the Lower Cretaceous of Montana. *Am. Mus. Novit.* **3240**, 1-27 (1998).
4. Lü, J.C. et al. Chicken-sized oviraptorid dinosaurs from central China and their ontogenetic implications. *Naturwissenschaften* **100,** 165–175 (2013).
5. Xu, X., Tan, Q. W., Wang, J. M., Zhao, X. J. & Tan, L. A gigantic bird-like dinosaur from the Late Cretaceous of China. *Nature* **447**, 844–847 (2007).
